# Cyclic Stability of Two-Way Shape Memory Effect in Aged Ni_50.3_Ti_32.2_Hf_17.5_ Polycrystals after Various Thermomechanical Treatments

**DOI:** 10.3390/ma16186175

**Published:** 2023-09-12

**Authors:** Elena Y. Panchenko, Anton I. Tagiltsev, Ekaterina E. Timofeeva, Yuriy I. Chumlyakov, Ekaterina S. Marchenko

**Affiliations:** Laboratory for Physics of High-Strength Crystals, Siberian Physical-Technical Institute, Tomsk State University, Lenina Str. 36, 634050 Tomsk, Russia; panchenko@mail.tsu.ru (E.Y.P.); katie@mail.tsu.ru (E.E.T.); chum@phys.tsu.ru (Y.I.C.); 89138641814@mail.ru (E.S.M.)

**Keywords:** martensitic transformation, NiTiHf, stress-induced martensite aging, two-way shape memory effect, cyclic stability

## Abstract

In the present paper, the cyclic stability of the high-temperature two-way shape memory effect was studied in high-strength Ni_50.3_Ti_32.2_Hf_17.5_ polycrystals after various thermomechanical treatments—training (thermocycling under stress) and stress-induced martensite aging. The effect of training and stress-induced martensite aging on the microstructure, the two-way shape memory effect, and its cyclic stability was determined. It was found out that both thermomechanical treatments induce the high-temperature two-way shape memory effect at T > 373 K, with a strain of 1.5% in tension. The influence of cyclic tests (up to 100 stress-free cycles of cooling/heating) on the two-way shape memory effect strain, the transformation temperatures, and the microstructure was established. Different degradation mechanisms of the two-way shape memory effect were established after thermocycling and stress-induced martensite aging.

## 1. Introduction

The NiTiHf system is already known as a reliable, high-strength, high-temperature material with a wide range of necessary properties and characteristics for use in various fields of activity such as actuators, sensors, complex working mechanisms, and dampers [1,2,3,4,5].

One of the functional properties that expands the possibilities of the practical application of shape memory alloys is the two-way shape memory effect (TWSME) [2]. A material with the TWSME is more promising for use as an actuator, since there is no need to use external stresses to return the working element to its original position, which simplifies the design of working devices. It should be noted that one of the main requirements for practical application is the cyclic stability of the functional properties during long-term operation, without any significant degradation. Therefore, the induction of the cyclically stable TWSME in NiTiHf alloys is an actual problem. In this respect, a NiTiHf alloy with the addition of the Hf element, from 10% to 20%, is under scientific interest because it makes it possible to achieve an increase in the transformation temperatures above 100 °C (373 K) and implement the high-temperature TWSME in alloys without facing bad plasticity due to the high content of Hf [1,6].

Two methods were used to induce the TWSME in NiTiHf systems: the thermal cycling of the material through the temperature range of the thermoelastic martensitic transformations (MTs) under the applied external stresses (training) and stress-induced martensite aging (SIM-aging). It is known from the literature [7] that training is mostly used to obtain the TWSME in NiTiHf alloys. Such treatment, which leads to the formation of a large number of dislocations and residual martensite, makes it possible to obtain the TWSME with a reversible strain of up to 1.3–1.5% in tension in polycrystals with a small grain size (d < 1 µm) [7]. However, it was shown in other shape memory alloys (NiFeGaCo and CoNiAl) that SIM-aging is more effective at obtaining the high cyclic stability of the TWSME with a large reversible strain of up to 9% [8,9]. During SIM-aging, the redistribution of point defects and atoms of different kinds happens in accordance with the martensite symmetry, and, thus, martensite stabilization occurs. In our previous work [10], it was shown that due to SIM-aging it is possible to obtain a TWSME of up to 2.3% in aged Ni_50.3_Ti_32.2_Hf_17.5_ and Ni_50.2_Ti_37.3_Hf_12.5_ alloys, which exceeds the literature data obtained on the polycrystals of the Ni_50.3_Ti_29.7_Hf_20_ alloy after extrusion.

However, there has not been an in-depth study of SIM-aging and training on the TWSME and its cyclic stability in a high-temperature NiTiHf system nor a comparison of both methods to determine the most optimal thermomechanical treatment in order to obtain a stable TWSME with the maximum strain. Therefore, the aim of the present work is to find out whether the method of inducing the high-temperature TWSME (training or SIM-aging) affects its cyclic stability, martensite morphology, and microstructure in the polycrystalline Ni_50.3_Ti_32.2_Hf_17.5_ (at.%) alloy.

## 2. Materials and Methods

Polycrystals of Ni_50.3_Ti_32.2_Hf_17.5_ alloy (at.%), obtained by electric arc melting from high-purity components (99.99%), were used in the present work. The samples for tension were cut to the size of 20 × 1.5 × 2.5 mm^3^ in the dog-bone form by using the electro-discharge machine and were, subsequently, mechanically ground and electrolytically polished. The average grain size in polycrystals was ~36 µm, and no significant change was observed after additional thermal treatments. To date, polycrystals after extrusion with a small grain size have been mostly studied [11,12], whereas polycrystals with a large grain size d > 10 µm, in which the texture is absent or has a weakly pronounced shape, have been poorly studied.

The chemical composition of the material was controlled by using an XRF-1800 X-ray fluorescence wave-dispersion spectrometer (Shimadzu, Kyoto, Japan). It was experimentally shown that the chemical composition of Ni_50.3_Ti_32.2_Hf_17.5_ (at.%) polycrystals on average over a surface of 10 mm^2^ corresponds to the nominal, within the measurement error of 5% from the measured value. The chemical composition was chosen based on [13], which shows that the addition of Hf from 10 to 20 at.% contributes to a significant increase in the characteristic temperatures of MT (20–25 K per 1 at.%).

The material was studied in the following states: (I) polycrystals after melting were aged in austenite in stress-free state at 773 K for 3 h, followed by slow cooling; (II) (I) + SIM-aging (300 MPa, 428 K, and 12 h); (III) (I) + training at applied stress of 300 MPa (thermal cycling for 10 cycles through the MT interval, which corresponds to a total time of 12 h).

Thermomechanical treatments were carried out using a dilatometer (IMRS-1, Microsplav, Tomsk, Russia).

Mechanical tests were carried out on a specially designed installation for measuring SME at cooling/heating cycles under constant tensile stress (IMRS-1, Microsplav, Tomsk, Russia). The measurement error was ~0.3% for deformation and 3K for temperature. The microstructure of crystals was obtained using a transmission electron microscope Hitachi HT-7700 (Hitachi, Tokyo, Japan). The electron microscopic studies were carried out on the equipment of the Krasnoyarsk Regional Center for Collective Use SB RAS.

The aging temperature in austenite, 773 K for 3 h, was chosen based on [13,14,15], where it was shown that during this heat treatment the nanoscale dispersed particles of the H-phase are precipitated, strengthening the material and increasing the characteristic temperatures of MT [14,15,16]. Transformation temperatures of aging in austenite in stress-free state, at 773 K for 3 h crystals (initial polycrystals), were obtained using differential scanning calorimeter DSC 404F1 Pegasus (NETZSCH, Selb, Germany) with a cooling/heating rate of 10 K/min.

The regime of SIM-aging was as follows: the sample was preloaded to 300 MPa and cooled below the martensite finish temperature M_f_ so that an oriented martensite grew in the entire volume of the material. Then, the heating was carried out to the austenite start temperature A_s_, in order to reach the highest possible holding temperature without the implementation of reverse MT. After that, the material was kept for 12 h under these conditions and then, subsequently, heated to a temperature above austenite finish temperature A_f_, so the reverse MT would occur. The SIM-aging time was selected on the basis of previous work [10] so that no changes in the “deformation-temperature” curve were observed during the last hour of SIM-aging.

The number of training cycles was selected in such a way that the total time spent by the material under an applied stress of 300 MPa corresponded to the time of SIM-aging.

The stresses for both thermomechanical treatments to induce TWSME (training and SIM-aging) were chosen in such a way that the material did not collapse and had the largest reversible strain and the minimum irreversible strain (0.3–0.5%).

## 3. Results

**Figure 1:**
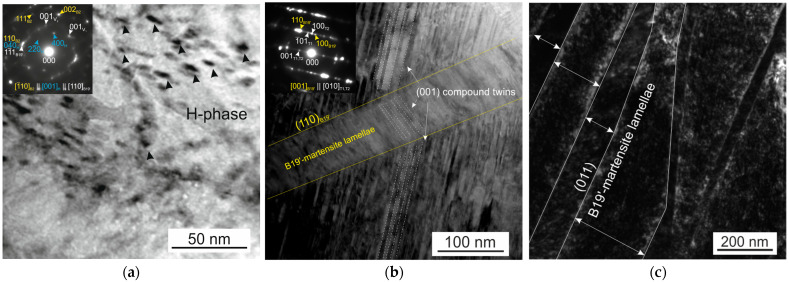
Bright-field images with corresponding SAEDPs of Ni_50.3_Ti_32.2_Hf_17.5_ polycrystals aged in austenite at 773 K for 3 h presenting H-phase dispersed particles (**a**), internally twinned martensite lamellae (**b**), and the width of martensite lamellae (**c**).

It was shown by electron microscopic studies that Ni_50.3_Ti_32.2_Hf_17.5_ (at.%) polycrystals aged at 773 K for 3 h contain nanoscale dispersed H-phase particles with sizes of 10–15 nm. Figure 1 shows the microstructure of the initial polycrystals aged at 773 K for 3 h before thermomechanical treatments. On the selected area electron diffraction pattern (SAEDP), the reflexes of the B2 matrix and characteristic reflexes 1/2<111>_B2_, which indicate the presence of H-phase particles, are clearly shown [13,14,15]. The H-phase particles have a face-centered orthorhombic lattice with parameters a = 4a_0_, b = 2$\sqrt{2}$a_0_, and c = 6$\sqrt{2}$a_0_ [13,14,15]. It was shown that the microstructure is characterized by the presence of wide martensitic lamellae with dimensions of 50–300 nm, which contain internal compound twins (001)_B19′_ (Figure 1b,c). These martensitic lamellae are twinned by {011} type I, and the dispersed particles of the H-phase are completely embedded in the martensite variants, which is consistent with [17], where internally twinned lamellae of B19′-martensite were also found.

**Figure 2:**
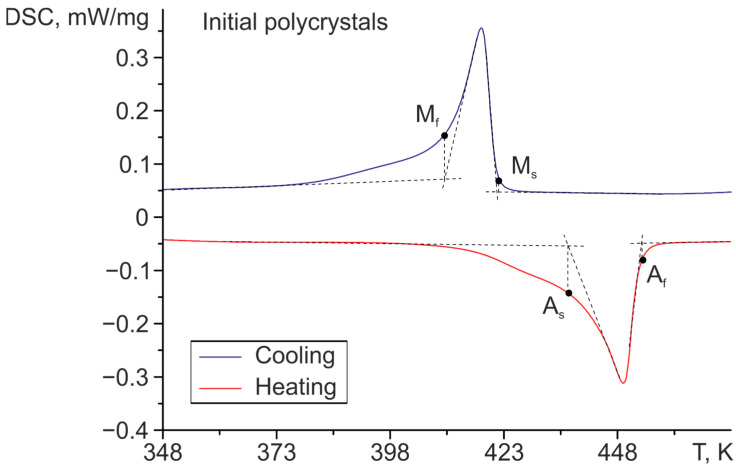
Differential scanning calorimetry for Ni_50.3_Ti_32.2_Hf_17.5_ polycrystals aged in austenite at 773 K for 3 h (initial polycrystals).

Before applying any thermomechanical treatment to the initial polycrystals, the transformation temperatures were obtained (Figure 2), which were found to be in accordance with the literature [1,2,3,4,5].

**Figure 3:**
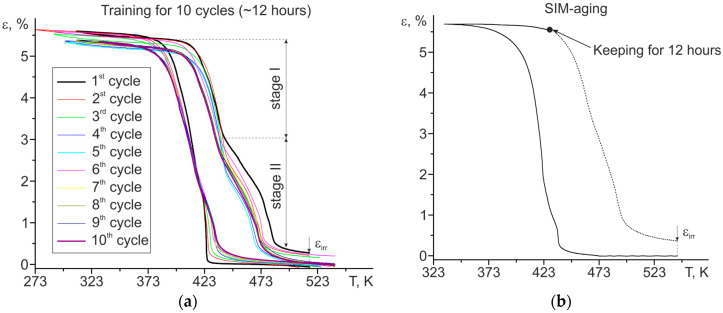
The strain–temperature curves for aged Ni_50.3_Ti_32.2_Hf_17.5_ polycrystals during (**a**) training and (**b**) SIM-aging.

Initial polycrystals aged at 773 K for 3 h were subjected to thermomechanical treatments in accordance with Section 2—thermal cycling under stress of 300 MPa (10 cycles~12 h) (Figure 3a) and SIM-aging at T = 428 K, 300 MPa, and 12 h (Figure 3b).

During training, at cooling/heating cycles under stress of 300 MPa, the reversible strain was 5.3% (Figure 3a), which is in accordance with the literature [18]. The accumulation of an irreversible deformation ε_irr_ of 0.3% occurred during training. This irreversibility is associated with plastic deformation and the appearance of residual martensite. At the same time, there was a staging on the curve during the training process related to the macrolocalization of the deformation in one part of the sample.

The strain–temperature curve during SIM-aging is shown in Figure 3b. The solid part of the curve corresponds to the initial state of the polycrystals, while the dotted part of the curve is the heating after SIM-aging and responds to the already-SIM-aged state of the polycrystals. It should be noted that the curve also shows an irreversibility of 0.35%, as in the samples after training, which may be caused by the presence of both the stabilized martensite and the partial plastic deformation occurring near the grain boundaries.

The optical studies of the samples’ working surface were carried out at room temperature after training and SIM-aging. It was found that, after SIM-aging, the working surface of the material is homogeneous—a relief associated with the formation of oriented martensite during cooling was observed throughout the material’s volume. On the contrary, after training, the surface of the working part of the sample was inhomogeneous—a localized area consisting of a mixture of residual martensite and plastic deformation bands was optically observed. Such localization of the deformation may be the reason for the stages of the ε(T) curves during training. It was found that neither training nor SIM-aging affect the grain size or H-phase particles in aged Ni_50.3_Ti_32.2_Hf_17.5_ polycrystals.

**Figure 4:**
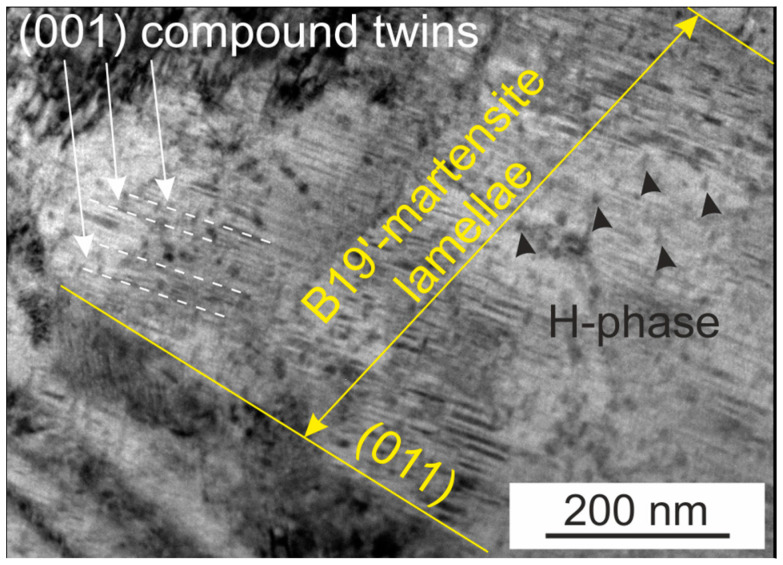
Bright-field image of aged Ni_50.3_Ti_32.2_Hf_17.5_ polycrystals after SIM-aging.

**Figure 5:**
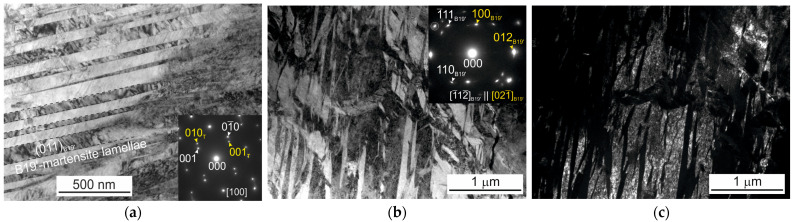
Bright-field images with corresponding SAEDPs of aged Ni_50.3_Ti_32.2_Hf_17.5_ polycrystals after training from two parts of the sample (**a**) without traces of plastic deformation, [100]_B19′_ zone axis, and (**b**) with mixture of several intersecting split martensitic systems. (**c**) Dark-field image of split martensite systems taken in circled reflex of (**b**).

Figure 4 and Figure 5 show the microstructure of polycrystals after SIM-aging and training, respectively. Since polycrystals are characterized by a high temperature of martensitic transformation, starting at M_s_ > 400 K, the material was in a martensite during the electron microscopic studies, and thermal-induced martensite is observed in both figures.

It was experimentally found that, after SIM-aging, a high density of nanotwins was observed along the planes of the (001) B19′-martensite. It should be noted that, after SIM-aging, the width of the B19′-martensite lamellae increased to 500 nm (Figure 4), compared with the initial state. The wide lamellae in Figure 4 are the witness of the oriented growth of thermally induced martensite after SIM-aging.

A different morphology of thermal-induced martensite was observed after training (Figure 5), compared with the SIM-aged crystals. Foils for the electron microscopic studies were cut from two parts of the sample—a strongly deformed particle of the sample, in which the localization of residual deformation was observed, and a second part of the sample, where, macroscopically, there were no traces of plastic deformation. It is assumed that in the clean area of the sample after training, type I {011} twins with a lamella width of up to 100 nm were found, inside of which thinner compound twins were presented (Figure 5a). Such a microstructure of martensite differs little from the one, which was observed in the initial crystals. However, a complex microstructure was found in the deformed part of the sample (Figure 5b,c), which is a mixture of several “split” martensitic systems intersecting each other. Such behavior may be a consequence of the high density of dislocations and the presence of residual martensite and internal stresses that induce the formation of various martensite variants, in contrast with SIM-aged crystals (Figure 4 and Figure 5).

**Figure 6:**
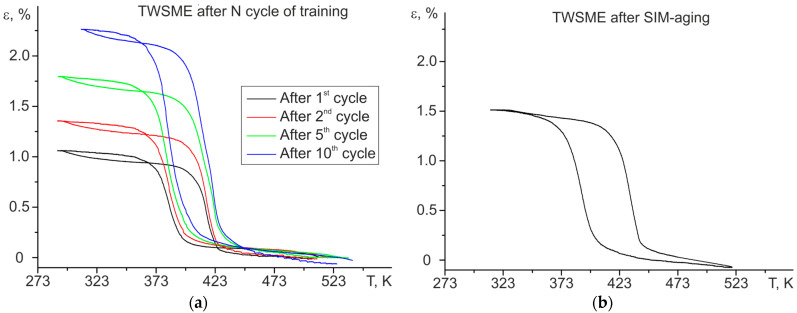
The strain–temperature curves of TWSME for aged Ni_50.3_Ti_32.2_Hf_17.5_ polycrystals after (**a**) N^th^ cycle of training and (**b**) SIM-aging.

Both regimes (training and SIM-aging) lead to the induction of a high-temperature TWSME at T > 373 K for stress-free cooling/heating cycles (Figure 6).

The value of the TWSME for stress-free cooling/heating cycles after training is determined by the number of thermal cycles under stress during training. The TWSME strain was equal to 1% after the 1st training cycle and increased to 2.3% after the 10th training cycle.

SIM-aging for 12 h led to a TWSME of 1.5%. It should be noted that, in our previous work on the same polycrystals, after SIM-aging a TWSME strain of up to 2.3% was obtained, which differs from the results shown in the present paper. Such behavior can be explained as follows. First, the maximum reversible strain is affected by the strong orientation dependence of the B2-B19′ transformation in polycrystals with a sufficiently large grain size. Therefore, if grains with a predominantly [001] orientation are presented in the sample, then the total transformation strain becomes significantly less, since along the [001] direction one of the lowest strain values is observed (2.92%) in tension, whereas along the [011] and [111] directions the transformation deformation is higher by 2.5–3.5 times. Second, in the NiTiHf system, both in polycrystals and single crystals, the full lattice deformation resource is not realized, so only local regions are observed in which the deformation can reach theoretical values [19].

**Figure 7:**
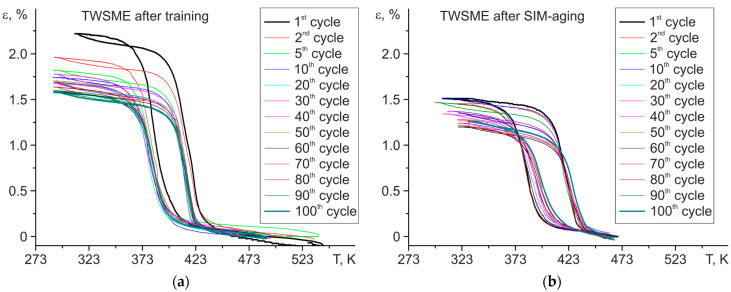
Cyclic stability of TWSME for aged Ni_50.3_Ti_32.2_Hf_17.5_ polycrystals after (**a**) 10 training cycles and (**b**) SIM-aging.

**Figure 8:**
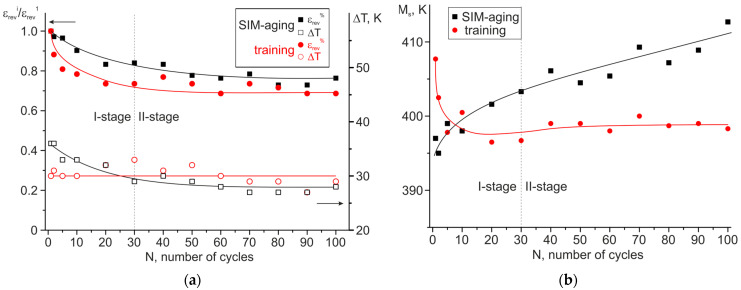
The dependence of (**a**) reversible strain change (presented in fractions as ε_rev_^i^/ε_rev_^1^), thermal hysteresis, and (**b**) M_s_ temperature on the number of cooling/heating cycles for aged Ni_50.3_Ti_32.2_Hf_17.5_ polycrystals after training and SIM-aging.

To estimate the stability of the functional properties of the TWSME, cycling (100 stress-free cooling/heating cycles) was carried out after training and SIM-aging (Figure 7). Using the curves ε(T), the change in reversible strain ε_rev_ (presented in a fraction as a ratio of the “i” cycle to the first cycle, ε_rev_^i^/ε_rev_^1^), thermal hysteresis ΔT, and M_s_ temperature were calculated depending on the number of cycles (Figure 8). Based on the dependence of the reversible strain ε_rev_, thermal hysteresis ΔT, and M_s_ temperature on the number of cycles, two stages of degradation can be distinguished, which is typical for shape memory alloys—the first stage of initial degradation (from 1 cycle to 30 cycles), at which the parameters of the TWSME significantly change, and the stage of cyclic stability (from 31 cycles to 100 cycles).

The following changes in the characteristics of the material’s functional properties were experimentally established. First, the reversible strain decreased at the first stage of degradation by 27% and in polycrystals after training and SIM-aging by 17%, respectively. During the second stage of degradation (from 31 to 100 cycles), the reversible strain practically did not change and was ~1.5% and ~1.2% for samples after training and after SIM-aging, respectively. Second, thermal hysteresis almost does not change in polycrystals after training and is ~30 K. After SIM-aging, thermal hysteresis was reduced by 20% in the first 30 cycles to ~27 K and did not change with a further increase in cycles. Third, in polycrystals after training, the M_s_ temperature decreased during cycling by 10 K for the first 30 cycles and then slightly changed. In polycrystals after SIM-aging, on the contrary, the M_s_ temperature increased by 10 K during the first 30 cycles and weakly increased by an additional 5 K from 31 to 100 cycles.

## 4. Discussion

Despite the different mechanisms of TWSME induction and its degradation, it was found that both methods of TWSME generation (training and SIM-aging) induce a stable high-temperature TWSME of up to 1.5% in tension in the heterophase polycrystals of the Ni_50.3_Ti_32.2_Hf_17.5_ alloy with a large grain size containing dispersed H-phase particles. A previous study [5] showed that the maximum strain of the TWSME caused by the trainings was ~1.5%, which was obtained in the Ni_50.3_Ti_29.7_Hf_20_ polycrystals after extrusion in the tension. The value of thermal hysteresis and the temperature intervals of MT slightly differed in both states.

The analysis of both thermomechanical treatments (training and SIM-aging) should start from the observed irreversible strain. On the one hand, in trained samples it is associated with plastic deformation and the appearance of residual martensite. There is a staging on the curve during the training process related to the macrolocalization of the deformation in one part of the sample (Figure 3a). On the other hand, in SIM-aged samples the irreversibility may be caused by the presence of both the stabilized martensite, which is verified by the widening of martensite lamellae up to 500 nm (Figure 4), and the partial plastic deformation occurring near the grain boundaries.

Electron microscopy studies and metallographic studies of the surface confirmed that the microstructure of samples after training and SIM-aging differs. After training, the two regions can be distinguished: the first one without any traces of plastic deformation and the second one with residual martensite and deformation bands. In the second region, the high density of dislocations and the presence of residual martensite and internal stresses that induce the formation of various martensite variants are the reasons for the complex microstructure observed in polycrystals after training in Figure 5b,c. A similar microstructure was earlier observed on polycrystalline tubes of the Ni_50.3_Ti_29.7_Hf_20_ alloy [2]. Thus, thermoelastic MT occurred in a different way in these two regions, and the staging on the curve is the evidence of such a behavior (Figure 3). On the contrary, SIM-aged crystals, compared to polycrystals aged in austenite at 773 K for 3 h, possess the same microstructure without a high density of dislocations or regions with macrolocalization of the plastic deformation (Figure 4).

The above changes are associated with different degradation mechanisms in polycrystals, depending on the thermomechanical treatment. In polycrystals after training, a microstructure consisting of a large number of dislocations and residual martensite was formed, which induced the formation of various intersecting variants of martensite, as shown in Figure 5b,c. In [20,21], the appearance of a large number of dislocations near the residual martensite during thermal cycling was detected on Ni-rich NiTi alloys. The main part of the dislocations formed during the reverse movement of the interphase boundary. Therefore, in the samples after training, the presence of residual martensite led to the formation of a large number of dislocations in the process of the repeated movement of the interphase boundary. These newly appeared dislocations caused the pinning of residual martensite during cycling.

On the other hand, the internal stresses <Δσ_in_> formed at training relax during subsequent cooling/heating cycles due to the presence of pinned residual martensite as well as due to the possible dislocations’ annihilation, which is caused by a significant increase in their density. The combination of these factors led to the sharp drop in the M_s_ temperature during cycling at stage I due to the relaxation of internal stresses, which indicates an austenite stabilization (Figure 7 and Figure 8). As long as the microstructure became stabilized, then the further stress-free thermal cycling did not lead to a change in the TWSME parameters.

In polycrystals after SIM-aging, the TWSME was formed due to a different mechanism, in contrast to training, namely, the martensite stabilization, in which the chemical and mechanical contributions can be distinguished [22]. Chemical stabilization occurs due to the redistribution of point defects and atoms and the changes in their short-range order, in accordance with the martensite symmetry. In turn, mechanical stabilization is associated with the pinning of twin/interphase boundaries by dislocations and point defects. Additionally, during prolonged exposure in martensite under stress at elevated temperature, the change (increase) in coherence between the H-phase particles and martensite may occur, which, in turn, leads to a mismatch between the particles and austenite and contributes to the appearance of internal stresses in the material; however, the confirmation of this fact requires additional research. Previous studies [23] showed that the coherency of H-phase particles plays an important role in controlling both transformation temperatures and stresses.

The above-mentioned factors related to TWSME induction result in different degradation mechanisms during cycling in SIM-aged polycrystals, compared with the trained polycrystals. The contribution of both the chemical martensite stabilization and the change in coherency between the dispersed nanosized H-phase particles and the matrix into the internal stresses <Δσ_in_>, causing TWSME, remains almost the same during cycling. The reason for such behavior is that neither chemical stabilization nor coherency should not change during thermal cycling, having a non-mechanical nature of appearance. At the same time, the residual martensite formation appears during cycling, and it becomes pinned by the newly appeared dislocations during the reverse motion of the interphase boundary. This indicates the decrease in TWSME strain with each cycle, as in the trained crystals, since the overall volume of the matrix undergoing the MT reduces. In SIM-aged polycrystals, the further mechanical stabilization of martensite occurs during cycling, resulting in the easier formation of martensite lamellae, an increase in the Ms temperature with the growth of the cyclic number, and a slight increase in MT temperature intervals (by 5–7 K). Thus, the internal stresses <Δσ_in_> in SIM-aged polycrystals do not decrease with cycling. That is why the sharp increase in the M_s_ temperature during thermal cycling is observed with an increase in the number of cycles (Figure 7). In contrast, the relaxation of internal stresses <Δσ_in_> in trained polycrystals leads to the decrease in Ms temperature because of austenite stabilization.

## 5. Conclusions

In the current work, the cyclic stability of the TWSME in Ni_50.3_Ti_32.2_Hf_17.5_ polycrystals with a large grain size after SIM-aging and training (thermocycling under stress) is investigated.

It is experimentally established that both thermomechanical treatments induced a stable high-temperature TWSME at T > 373 K, with a reversible strain of up to 1.5% in tension in heterophase Ni_50.3_Ti_32.2_Hf_17.5_ polycrystals. The microstructure of polycrystals, the mechanisms of the TWSME, and the degradation mechanisms of the TWSME (during 100 cooling/heating cycles) strongly depend on the thermomechanical treatment.

After SIM-aging, TWSME is formed due to several factors: the chemical and mechanical stabilization of martensite, with an additional possible change in coherence between the dispersed particles of the H-phase and the matrix. The degradation (a decrease in reversible strain and an increase in the characteristic temperatures of the MT) of the TWSME during 100 cooling/heating cycles occurs due to the formation of residual martensite. However, internal stresses (caused by a coherency change between the dispersed particles and the matrix and by martensite stabilization), which do not decrease because of the non-mechanical nature, leads to the formation of oriented martensite and, consequently, to the increase in the M_s_ temperature and the slight increase in the MT temperature intervals (by 5–7 K).

In turn, training (thermocycling under stress) leads to a complex internal microstructure—several “split” martensitic systems intersecting each other are observed—due to the high density of dislocations. In these samples, the TWSME occurs due to a large amount of residual martensite and the presence of a high density of dislocations, which leads to the appearance of internal stresses. During TWSME cycling, the relaxation of internal stresses occurs due to the presence of residual martensite as well as due to the possible dislocations’ annihilation, which is caused by a significant increase in their density during the repeated movement of the interphase boundary, which indicates an austenite stabilization.

## Data Availability

The data presented in this study are available on request from the corresponding author.
